# Epidemiological Profile, Spatial Dynamics, and Survival Outcomes of HIV/AIDS Patients in Brazil (2014–2024): A Nationwide Study of AIDS-stage Notification and Mortality

**DOI:** 10.1007/s10900-026-01614-6

**Published:** 2026-08-06

**Authors:** Bruno Almeida-Silva, Claudeir Dias da Silva-Junior, Ronald Rodrigues de Moura, Natalia Pereira da Silva, Bruno Rodrigo Assunção, Houemakou Rimaud Djidonou

**Affiliations:** 1 Federal University of Pernambuco, Recife, Pernambuco Brazil; 2 Translational Research Laboratory, Instituto de Medicina Integral Prof. Fernando Figueira (IMIP), Recife, Pernambuco Brazil; 3 Institute for Maternal and Child Health, IRCCS Burlo Garofolo, Trieste, Italy

**Keywords:** HIV, AIDS-stage notification, Spatial analysis, Survival analysis, Brazil

## Abstract

Despite universal access to combined antiretroviral therapy in Brazil, substantial inequalities persist in the HIV/AIDS epidemic. This nationwide retrospective study analyzed 753,071 notifications from the Brazilian Notifiable Diseases Information System (SINAN) between 2014 and 2024 to characterize the epidemiological profile, temporal trends, spatial distribution, factors associated with AIDS-stage notification, and predictors of AIDS-related mortality. HIV infection notifications accounted for 491,976 records, while AIDS case notifications accounted for 261,095. HIV infection notifications were concentrated among young adults, particularly men aged 20–29 years, whereas AIDS case notifications were more frequent in older age groups. Spatial analysis showed significant positive autocorrelation of smoothed municipal notification rates (Moran’s I = 0.2306; *p* < 0.001), with significant High-High clusters mainly in the South and Southeast regions. In the multivariable logistic regression model, older age was strongly associated with AIDS-stage notification, reaching the highest odds among individuals aged 60 years or older (AOR = 2.03; 95% CI: 1.96–2.09), while higher education was associated with lower odds. In the Cox model, age at diagnosis was the main predictor of mortality, with increased risk among individuals aged 30–49 years (AHR = 1.42; 95% CI: 1.36–1.49) and 50 years or older (AHR = 1.95; 95% CI: 1.86–2.05). Lower education and injecting drug use were also strongly associated with AIDS-related mortality. These findings indicate that, although AIDS case notifications have declined over time, AIDS-stage notification and mortality remain concentrated in socially and geographically vulnerable groups.

## Introduction

Despite significant advances in the control of human immunodeficiency virus (HIV) infection, AIDS remains a major global public health concern. Since the beginning of the epidemic, HIV has claimed approximately 42.3 million lives, and in 2023 alone, an estimated 630,000 HIV-related deaths occurred worldwide. In response to this scenario, the World Health Organization (WHO) has promoted global strategies focused on prevention, early diagnosis, treatment, service delivery, and monitoring, while also addressing human rights issues such as stigma and discrimination, with the goal of ending AIDS as a public health threat [1].

A major milestone in the history of HIV infection was the introduction of combined antiretroviral therapy (c-ART) in the late 1990s. This therapy substantially changed the prognosis of infected individuals, allowing longer survival and, consequently, better quality of life. Its implementation resulted in significant reductions in the incidence of opportunistic infections, as well as in hospitalization and mortality rates in different parts of the world [2, 3].

However, even with universal and free antiretroviral treatment in Brazil, AIDS-related deaths continue to occur. Factors such as advanced-stage diagnosis, low adherence to HAART, drug toxicity, and the development of viral resistance still represent significant challenges for epidemic control [4]. Therefore, the analysis of HIV/AIDS mortality remains an essential tool for monitoring the progress of health policies and identifying the most vulnerable populations.

Brazil, a country of continental dimensions, has a heterogeneous AIDS epidemic, with different mortality patterns across its regions and states [5, 6]. Although the national mortality trend has declined in recent decades, this pattern is not uniform throughout the country [7]. Studies have shown that, while the South and Southeast regions have recorded decreasing mortality trends, the North and Northeast regions have shown increasing trends [5]. This regional disparity highlights the need for health policies that consider the substantial socioeconomic inequalities and unequal access to health services across the country.

The Brazilian epidemic has also changed in relation to the sociodemographic profile of the most affected populations. Temporal analyses indicate important changes in mortality according to sex, age group, and race/skin color [5, 6]. Mortality has increased in older age groups, particularly among individuals older than 50 years, and the proportional burden of deaths has grown among black and parda populations, pointing to persistent inequities in access to health care [5]. Given this complex and multifaceted scenario, further analysis of AIDS mortality trends in Brazil is essential to understand local dynamics and guide more equitable and effective public health actions [6].

Thus, this study proposes a comprehensive analysis of the HIV/AIDS epidemic in Brazil using recent national data, including the description of the epidemiological profile and temporal trends from 2014 to 2024, the investigation of spatial distribution to identify risk clusters, and the assessment of sociodemographic factors associated with AIDS-stage notification and their impact on survival.

## Methods

### Study Design and Data Source

This was a retrospective, population-based study using secondary data from the Brazilian Notifiable Diseases Information System (SINAN). Nationwide annual notification files in .dbc format were analyzed. Microdata were obtained from the public data repository of the Department of Informatics of the Brazilian Unified Health System (DATASUS), Ministry of Health. The study included datasets for AIDS cases (AIDABRxx.dbc) and HIV infection notifications (HIVABRxx.dbc) from January 1, 2014, to December 31, 2024.

### Exclusion Criteria

Records with notification dates outside the study period were excluded. For the multivariable logistic and Cox regression models, a complete-case approach was used, excluding records with missing information for any predictor included in the models, namely sex, age group, race/skin color, education, geographic region, and exposure category. For the survival analysis, records with missing AIDS diagnosis dates, unknown clinical outcomes, or outcomes other than “alive” or “AIDS-related death” were excluded. In addition, records with inconsistent death dates, including dates before the AIDS diagnosis date or after the end of follow-up, were treated as censored.

### Variables

The variables analyzed included sex, age, race/skin color, education level, exposure category, geographic region, year of notification, notification type, and survival status. Age was categorized into the following groups for the descriptive and logistic regression analyses: <13, 13–19, 20–29, 30–39, 40–49, 50–59, and 60 + years. For the survival analysis, age at AIDS diagnosis was categorized as < 30, 30–49, and 50 + years. Race/skin color was classified as white, black, parda, yellow, or Indigenous. Education was categorized as fundamental education or less, high school, and higher education. Exposure category included men who have sex with men (MSM), heterosexual exposure, injecting drug use, and vertical transmission. Geographic region was classified according to the official Brazilian Institute of Geography and Statistics (IBGE) macro-regional division as North, Northeast, Southeast, South, and Center-West.

The outcome of the logistic regression analysis was AIDS-stage notification, defined operationally as a record from the national AIDS case notification database. HIV infection notifications were used as the comparison group. This definition was based on the structure of the SINAN databases and does not correspond to a clinical or immunological definition based on CD4 count or viral load.

### Statistical Analysis

All analyses were performed using R version 4.5.1. Categorical variables were described using absolute and relative frequencies, while age was summarized using median and interquartile range. The distribution of notifications by age and sex was visualized using population pyramids based on the mean annual number of notifications, with error bars representing 95% confidence intervals. Temporal trends were displayed using line graphs.

For the spatial analysis, notification data were aggregated at the municipal level. Population estimates for 2021 from the Brazilian Institute of Geography and Statistics (IBGE) were used as denominators to calculate cumulative notification rates per 100,000 inhabitants. Because rates in small municipalities may be unstable, empirical Bayesian smoothing was applied. Global spatial autocorrelation was assessed using Moran’s I. Local Indicators of Spatial Association (LISA) were then calculated to identify statistically significant spatial clusters. Municipalities were classified as High-High hotspots when high smoothed notification rates were surrounded by neighboring municipalities with similarly high rates, and as Low-Low coldspots when low rates were surrounded by neighboring municipalities with similarly low rates. Spatial outliers were classified as High-Low or Low-High according to the relationship between each municipality and its neighbors. P-values from LISA analyses were adjusted using the Benjamini-Hochberg method.

Factors associated with AIDS-stage notification were evaluated using multivariable logistic regression. The model included sex, age group, race/skin color, education, geographic region, and exposure category, selected a priori based on epidemiological relevance. Adjusted odds ratios (AORs) and 95% confidence intervals (95% CIs) were estimated.

To evaluate mortality after AIDS diagnosis, survival analysis was conducted among AIDS case notifications. The outcome was time, in months, from the date of AIDS diagnosis to AIDS-related death. Individuals who were alive at the end of follow-up, had died from other causes, or had inconsistent death dates were censored on December 31, 2024. Survival probabilities were estimated using the Kaplan-Meier method, and curves were compared using the log-rank test. A multivariable Cox proportional hazards model was fitted to identify factors associated with AIDS-related mortality, estimating adjusted hazard ratios (AHRs) and 95% CIs. The proportional hazards assumption was assessed using Schoenfeld residuals. When evidence of violation of this assumption was observed, AHRs were interpreted as average associations over the follow-up period.

### Data and Code Availability

To ensure transparency and reproducibility, all R scripts used for data loading, processing, analysis, and visualization, together with package version information and the original raw .dbc files, are publicly available in the GitHub repository: https://github.com/Bruno-4lmeida/SINAN_HIV.

## Results

### Epidemiological Profile and Temporal Trends

From 2014 to 2024, a total of 753,071 notifications were analyzed, including 491,976 HIV infection notifications (65.33%) and 261,095 AIDS case notifications (34.67%). The median age of the overall population was 33 years (IQR: 26–43), with a lower median age among individuals notified with HIV infection (31 years; IQR: 25–41) than among those notified as AIDS cases (36 years; IQR: 28–46). Male individuals predominated in both groups, accounting for 71.01% of all notifications. The sociodemographic and epidemiological characteristics of the study population are presented in Table 1.

**Table 1:** Sociodemographic and epidemiological characteristics of HIV infection and AIDS case notifications in Brazil, 2014–2024

Variable/category	Total, *n* = 753,071	HIV, *n* = 491,976	AIDS, *n* = 261,095
Age, years, median (IQR)	33 (26 to 43)	31 (25 to 41)	36 (28 to 46)
Sex, female	218,157 (28.97)	141,415 (28.74)	76,742 (29.39)
Sex, male	534,729 (71.01)	350,384 (71.22)	184,345 (70.60)
Sex, missing/ignored	185 (0.02)	177 (0.04)	8 (0.00)
Age group, 13 to 19 years	34,936 (4.64)	27,926 (5.68)	7,010 (2.68)
Age group, 20 to 29 years	253,383 (33.65)	185,039 (37.61)	68,344 (26.18)
Age group, 30 to 39 years	215,685 (28.64)	136,562 (27.76)	79,123 (30.30)
Age group, 40 to 49 years	138,677 (18.41)	79,906 (16.24)	58,771 (22.51)
Age group, 50 to 59 years	74,148 (9.85)	41,185 (8.37)	32,963 (12.62)
Age group, 60 years or older	32,630 (4.33)	17,746 (3.61)	14,884 (5.70)
Age group, missing/ignored	3,612 (0.48)	3,612 (0.73)	0 (0.00)
Race/skin color, white	274,643 (36.47)	175,395 (35.65)	99,248 (38.01)
Race/skin color, black	85,157 (11.31)	57,266 (11.64)	27,891 (10.68)
Race/skin color, parda	333,827 (44.33)	218,301 (44.37)	115,526 (44.25)
Race/skin color, yellow	5,356 (0.71)	3,732 (0.76)	1,624 (0.62)
Race/skin color, Indigenous	2,642 (0.35)	1,834 (0.37)	808 (0.31)
Race/skin color, missing/ignored	51,446 (6.83)	35,448 (7.21)	15,998 (6.13)
Education, fundamental or less	215,300 (28.59)	128,469 (26.11)	86,831 (33.26)
Education, high school	229,258 (30.44)	155,144 (31.53)	74,114 (28.39)
Education, higher education	119,726 (15.90)	84,106 (17.10)	35,620 (13.64)
Education, missing/ignored	188,787 (25.07)	124,257 (25.26)	64,530 (24.72)
Region, North	79,893 (10.61)	54,531 (11.08)	25,362 (9.71)
Region, Northeast	174,146 (23.12)	115,281 (23.43)	58,865 (22.55)
Region, Southeast	288,515 (38.31)	186,244 (37.86)	102,271 (39.17)
Region, South	150,234 (19.95)	94,574 (19.22)	55,660 (21.32)
Region, Center-West	60,283 (8.00)	41,346 (8.40)	18,937 (7.25)
Exposure category, heterosexual	405,833 (53.89)	279,935 (56.90)	125,898 (48.22)
Exposure category, men who have sex with men	224,946 (29.87)	139,327 (28.32)	85,619 (32.79)
Exposure category, injecting drug user	12,517 (1.66)	6,769 (1.38)	5,748 (2.20)
Exposure category, vertical transmission	246 (0.03)	148 (0.03)	98 (0.04)
Exposure category, missing/ignored	109,529 (14.54)	65,797 (13.37)	43,732 (16.75)

Values are presented as n (%), unless otherwise indicated. Age is presented as median and interquartile range (IQR). Percentages were calculated within each notification group

**Fig. 1:**
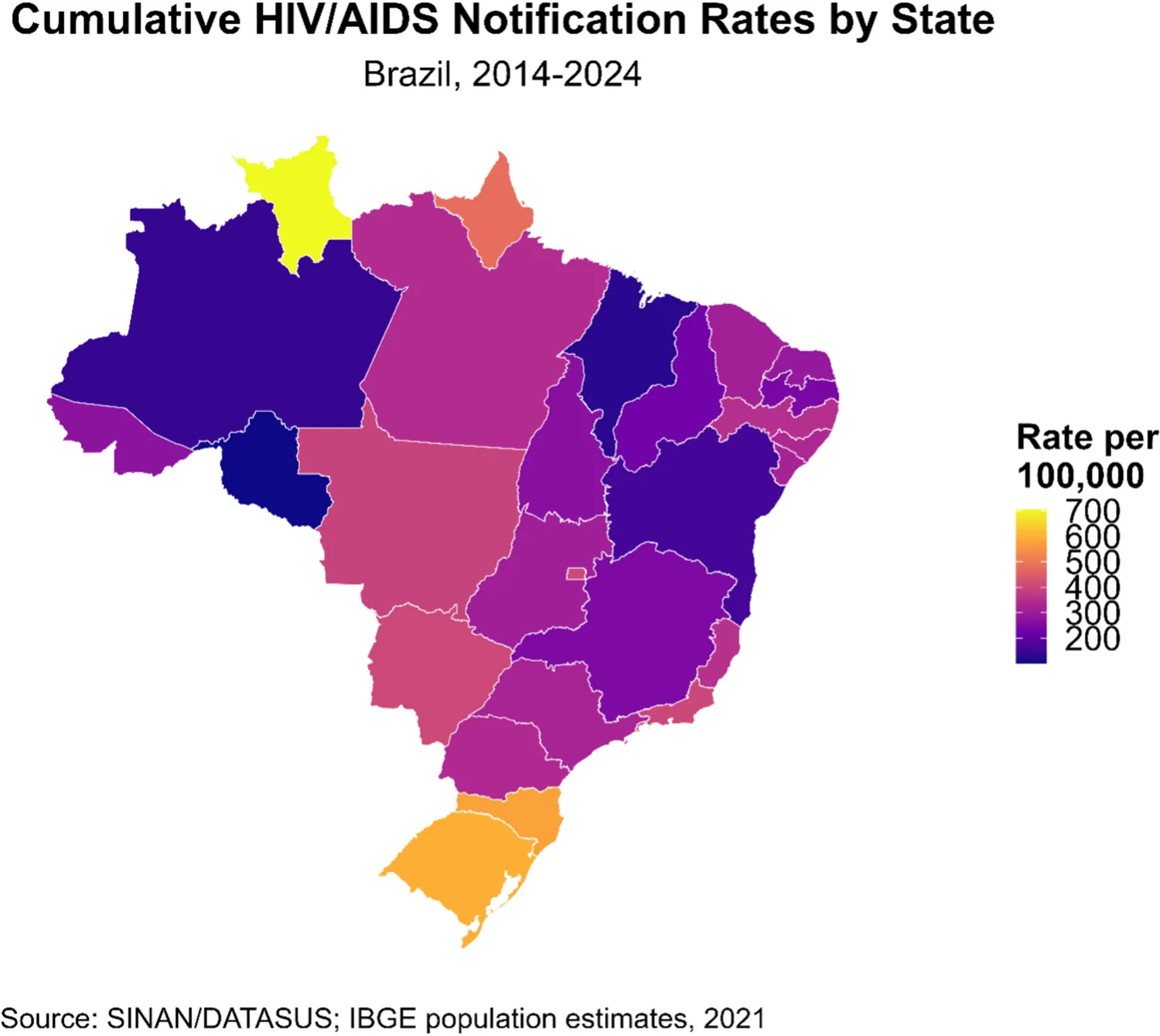
Cumulative notification rates of HIV/AIDS by Federative Unit (State). Rates are expressed per 100,000 inhabitants for the period of 2014–2024

The geographic distribution of cumulative notification rates per 100,000 inhabitants was heterogeneous across the country (Fig. 1). The highest rates were observed in the South region, particularly in Rio Grande do Sul, and in parts of the North region, especially Roraima and Amapá. States in the Southeast and Center-West regions showed intermediate rates, whereas the lowest rates were concentrated in the Northeast. This spatial pattern indicates that, although HIV/AIDS notifications were widely distributed across Brazil, their population-adjusted intensity was concentrated in specific regional pockets.

**Fig. 2:**
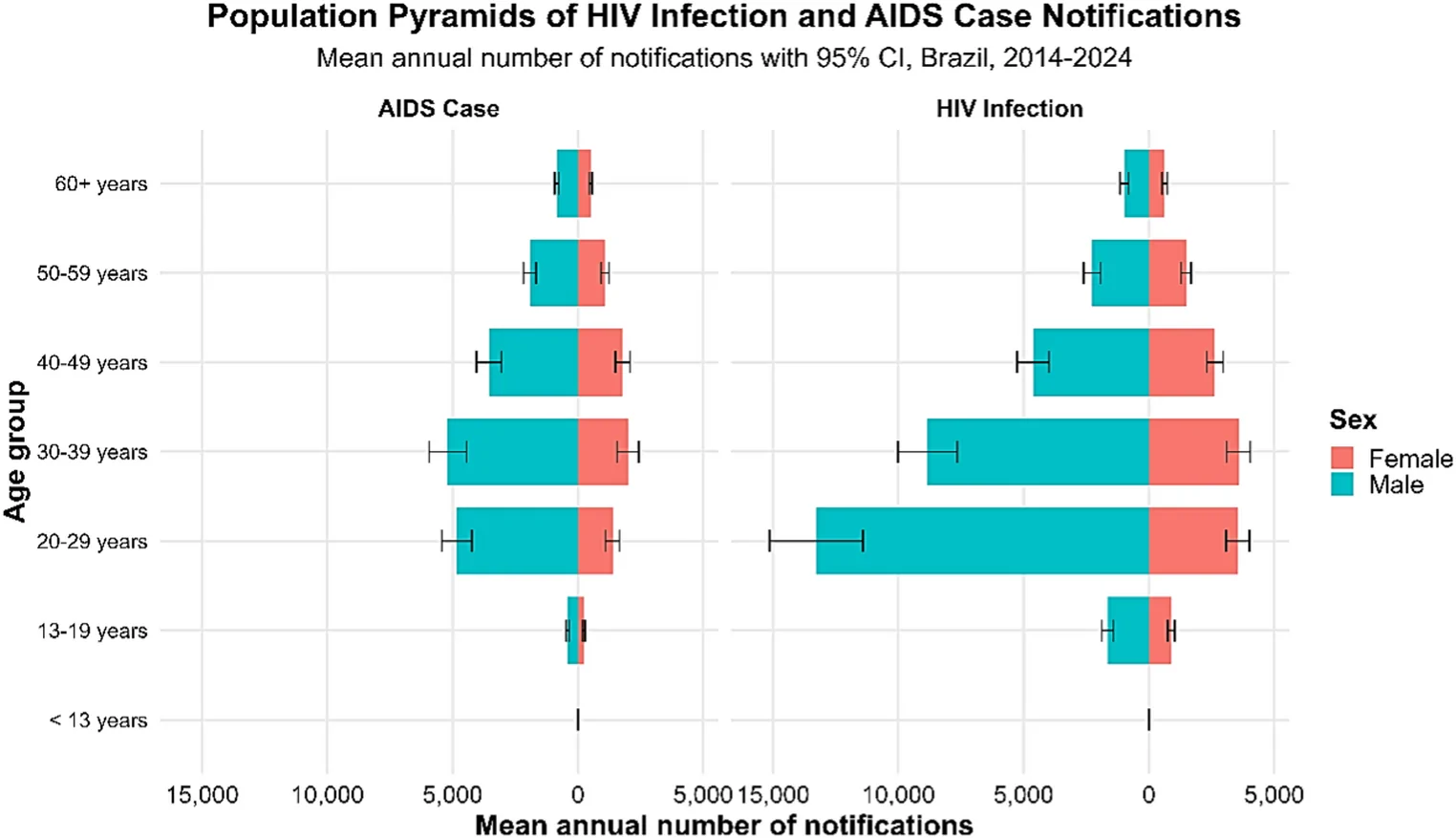
Population pyramids of HIV infection and AIDS case notifications by age group and sex in Brazil, 2014–2024. Bars represent the mean annual number of notifications, and error bars indicate 95% confidence intervals

The sociodemographic profile differed between HIV infection notifications and AIDS case notifications (Fig. 2). HIV infection notifications showed a younger age distribution, with the highest mean annual number of notifications in the 20–29-year age group, particularly among men. In contrast, AIDS case notifications were concentrated in older age groups, especially between 30 and 49 years. Male individuals accounted for most notifications in both groups, although this predominance was more pronounced among HIV infection notifications, particularly among young adults.

**Fig. 3:**
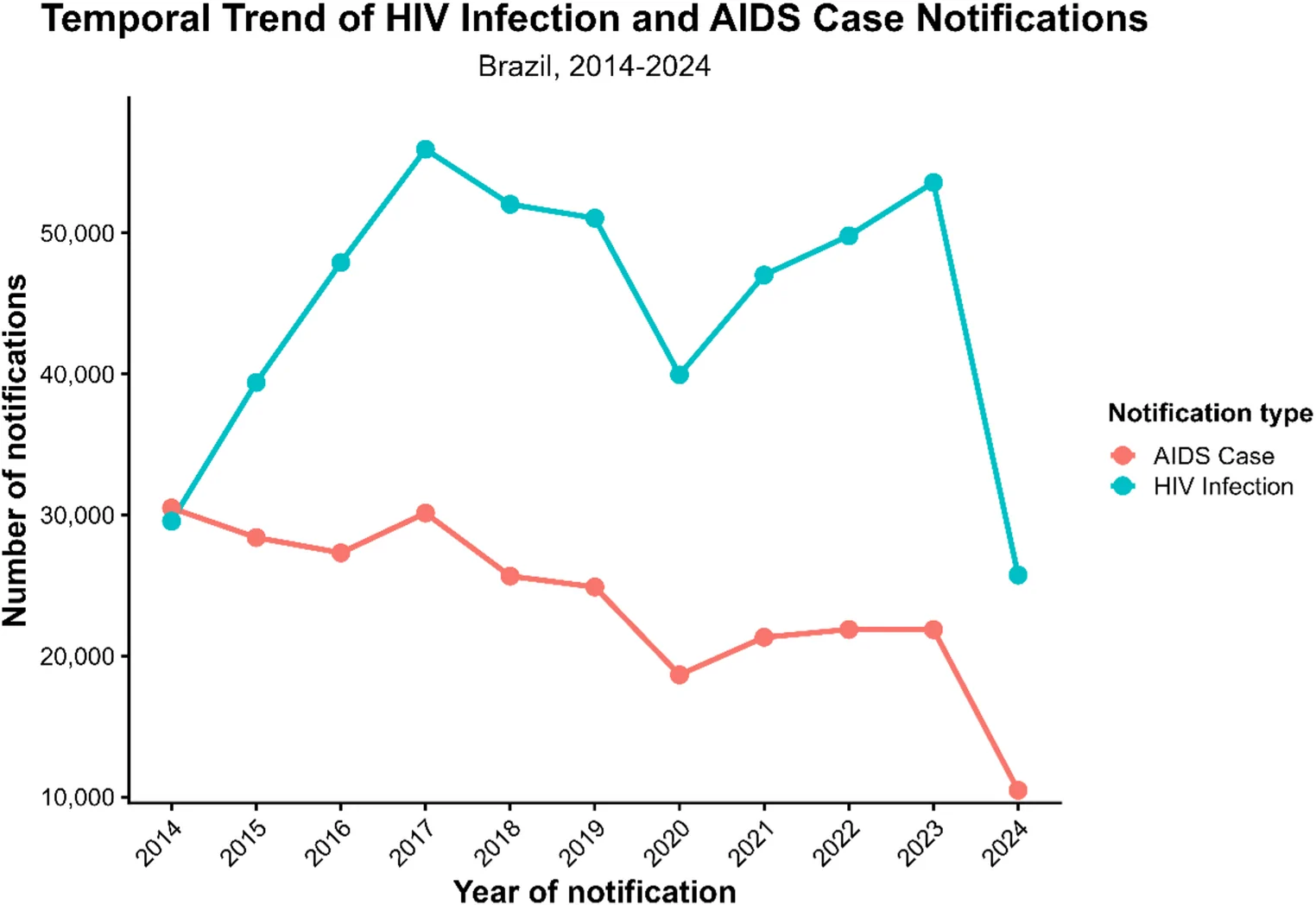
Temporal trend of HIV infection and AIDS case notifications. The lines represent the annual number of notifications in Brazil from 2014 to 2024

Between 2014 and 2024, HIV infection and AIDS case notifications showed distinct temporal patterns in Brazil (Fig. 3). HIV infection notifications increased markedly between 2014 and 2017, rising from approximately 30,000 to nearly 57,000 records. After this peak, a gradual decline was observed until 2019, followed by a more pronounced decrease in 2020, when notifications reached approximately 40,000 records. From 2021 onward, HIV infection notifications increased again, approaching pre-pandemic levels by the end of the study period.

In contrast, AIDS case notifications showed an overall declining pattern throughout the period. In 2014, slightly more than 30,000 AIDS cases were notified, followed by a progressive decrease in subsequent years. The lowest number of notifications occurred in 2020, with fewer than 20,000 records. After 2020, AIDS case notifications showed a modest recovery and then remained relatively stable, at approximately 22,000 annual records in the most recent years.

Together, these findings indicate a sustained reduction in AIDS case notifications over the last decade, while HIV infection notifications showed an initial increase, a pandemic-related decline in 2020, and a subsequent rebound from 2021 onward. This pattern is consistent with a surveillance scenario in which HIV detection remains active or increases, while progression to AIDS becomes less frequent, possibly reflecting expanded testing, earlier linkage to care, and broader access to combined c-ART.

**Fig. 4:**
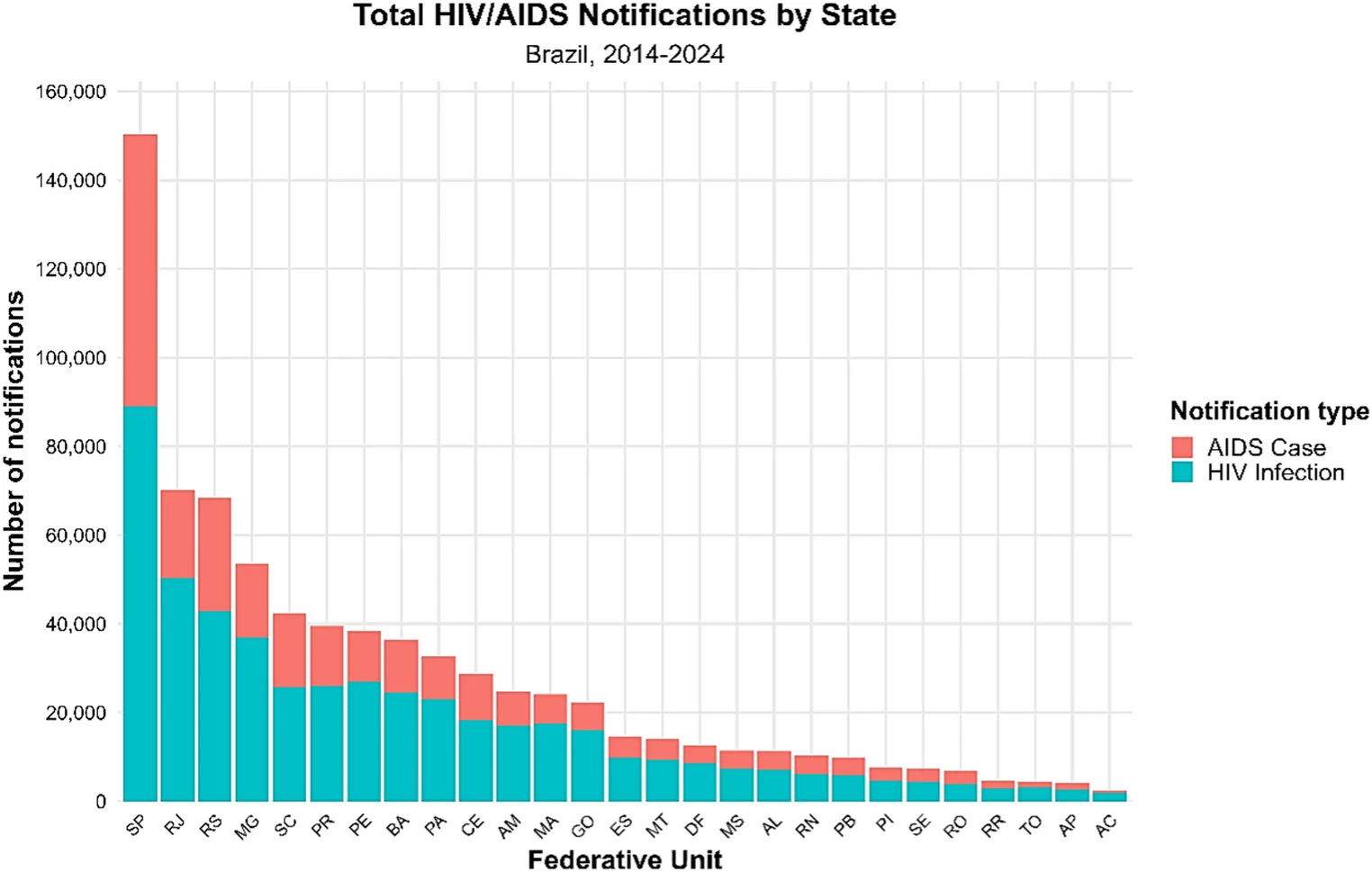
Absolute number of HIV infection and AIDS case notifications by Federative Unit in Brazil, 2014–2024. Bars represent the total number of notifications stacked by notification type. States are ordered according to total notification volume

Figure 4 shows the absolute number of HIV infection and AIDS case notifications by Brazilian Federative Unit from 2014 to 2024. In contrast to the population-adjusted rates shown in Fig. 1, the absolute notification burden was strongly concentrated in the most populous states. São Paulo had the highest number of notifications, exceeding 150,000 records, followed by Rio de Janeiro, Rio Grande do Sul, and Minas Gerais. These states accounted for the largest absolute numbers of both HIV infection notifications and AIDS case notifications. Conversely, less populous states, mainly located in the North and Center-West regions, showed the lowest absolute notification counts. This pattern indicates that, although population-adjusted rates highlight important regional pockets of higher intensity, the largest absolute volume of HIV/AIDS notifications remains concentrated in Brazil’s major demographic centers.

### Spatial Distribution and Clustering

Global Moran’s I indicated significant positive spatial autocorrelation of smoothed municipal notification rates (Moran’s I = 0.2306; *p* = 4.0 × 10⁻¹⁷), confirming that the geographic distribution of HIV/AIDS notifications was not random. The LISA analysis identified 211 municipalities with statistically significant local spatial autocorrelation after Benjamini-Hochberg adjustment, including 155 High-High hotspots and 56 Low-High spatial outliers. No significant Low-Low coldspots or High-Low outliers were detected.

**Fig. 5:**
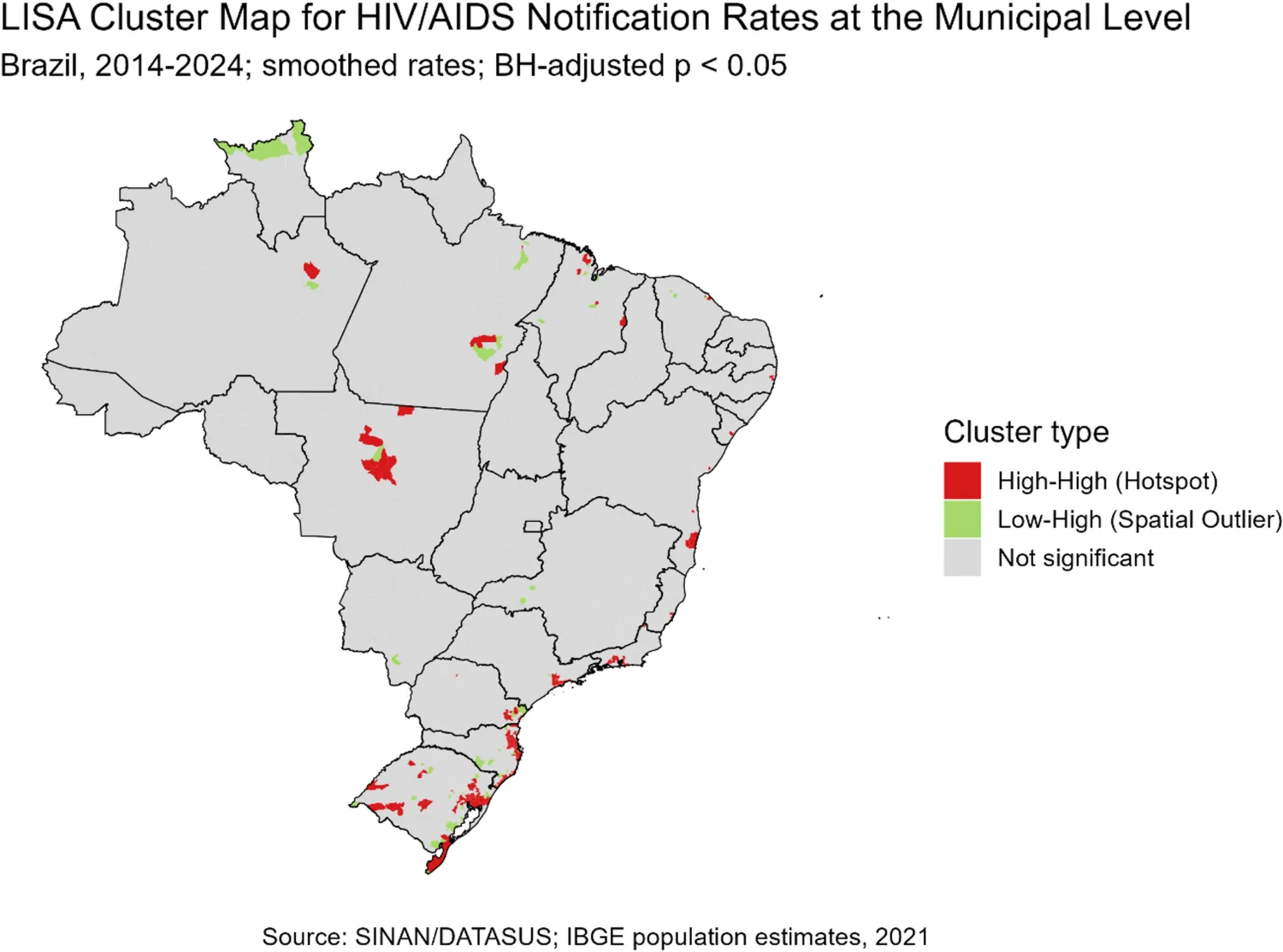
Spatial clusters of smoothed HIV/AIDS notification rates at the municipal level in Brazil, 2014–2024, based on Local Indicators of Spatial Association (LISA). The map displays statistically significant High-High hotspots and Low-High spatial outliers; no significant Low-Low coldspots or High-Low outliers were detected

High-High hotspots were mainly concentrated in the South and Southeast regions, particularly in Rio Grande do Sul, Santa Catarina, Rio de Janeiro, São Paulo, and Paraná, with additional clusters observed in selected municipalities from the Northeast, North, and Center-West regions. Low-High spatial outliers were also identified, representing municipalities with comparatively low smoothed notification rates surrounded by municipalities with higher rates (Fig. 5). The full list of municipalities included in statistically significant LISA clusters is provided in Supplementary Table S1.

### Factors Associated with AIDS-stage Notification

Among HIV/AIDS notifications recorded in Brazil between 2014 and 2024, the multivariable logistic regression model identified several factors associated with AIDS-stage notification, defined as records originating from the national AIDS case notification database rather than the HIV infection notification database (Table 2). Male sex was associated with lower odds of AIDS-stage notification compared with female sex (AOR = 0.93; 95% CI: 0.92–0.95; *p* < 0.001). Age showed a strong graded association with the outcome. Compared with individuals aged 20–29 years, adolescents aged 13–19 years had lower odds of AIDS-stage notification (AOR = 0.64; 95% CI: 0.62–0.66; *p* < 0.001), whereas older age groups showed progressively higher odds, reaching the highest estimate among individuals aged 60 years or older (AOR = 2.03; 95% CI: 1.96–2.09; *p* < 0.001).

**Table 2:** Multivariable logistic regression analysis of factors associated with AIDS-stage notification in Brazil, 2014–2024 (*N* = 500468)

Variable/category	AOR (95% CI)	*p*-value
Sex		
Female	Reference	
Male	0.93 (0.92–0.95)	< 0.001
Age group		
20–29 years	Reference	
13–19 years	0.64 (0.62–0.66)	< 0.001
30–39 years	1.48 (1.46–1.51)	< 0.001
40–49 years	1.82 (1.79–1.85)	< 0.001
50–59 years	1.93 (1.88–1.97)	< 0.001
60 + years	2.03 (1.96–2.09)	< 0.001
Race/skin color		
White	Reference	
Black	0.83 (0.81–0.85)	< 0.001
Indigenous	0.80 (0.72–0.88)	< 0.001
Parda	0.94 (0.92–0.95)	< 0.001
Yellow	0.77 (0.72–0.83)	< 0.001
Education		
Fundamental or less	Reference	
High school	0.86 (0.84–0.87)	< 0.001
Higher education	0.76 (0.75–0.78)	< 0.001
Region		
Southeast	Reference	
Center-West	0.90 (0.88–0.92)	< 0.001
North	0.90 (0.88–0.92)	< 0.001
Northeast	0.84 (0.83–0.85)	< 0.001
South	0.98 (0.96–1.00)	0.017
Exposure category		
Heterosexual	Reference	
Injecting drug user	1.63 (1.56–1.70)	< 0.001
Men who have sex with men (MSM)	1.27 (1.25–1.29)	< 0.001
Vertical transmission	1.22 (0.88–1.68)	0.222

Regarding race/skin color, black (AOR = 0.83; 95% CI: 0.81–0.85; *p* < 0.001), parda (AOR = 0.94; 95% CI: 0.92–0.95; *p* < 0.001), Indigenous (AOR = 0.80; 95% CI: 0.72–0.88; *p* < 0.001), and yellow individuals (AOR = 0.77; 95% CI: 0.72–0.83; *p* < 0.001) had lower odds of AIDS-stage notification than white individuals. Higher educational attainment was also associated with lower odds, particularly among individuals with higher education (AOR = 0.76; 95% CI: 0.75–0.78; *p* < 0.001). Compared with the Southeast region, all other regions showed lower odds of AIDS-stage notification, with the strongest inverse association observed in the Northeast (AOR = 0.84; 95% CI: 0.83–0.85; *p* < 0.001). By exposure category, injecting drug use (AOR = 1.63; 95% CI: 1.56–1.70; *p* < 0.001) and being a man who has sex with men (AOR = 1.27; 95% CI: 1.25–1.29; *p* < 0.001) were associated with higher odds of AIDS-stage notification compared with heterosexual exposure. Vertical transmission was not significantly associated with the outcome (AOR = 1.22; 95% CI: 0.88–1.68; *p* = 0.222).

### Survival Analysis

**Fig. 6:**
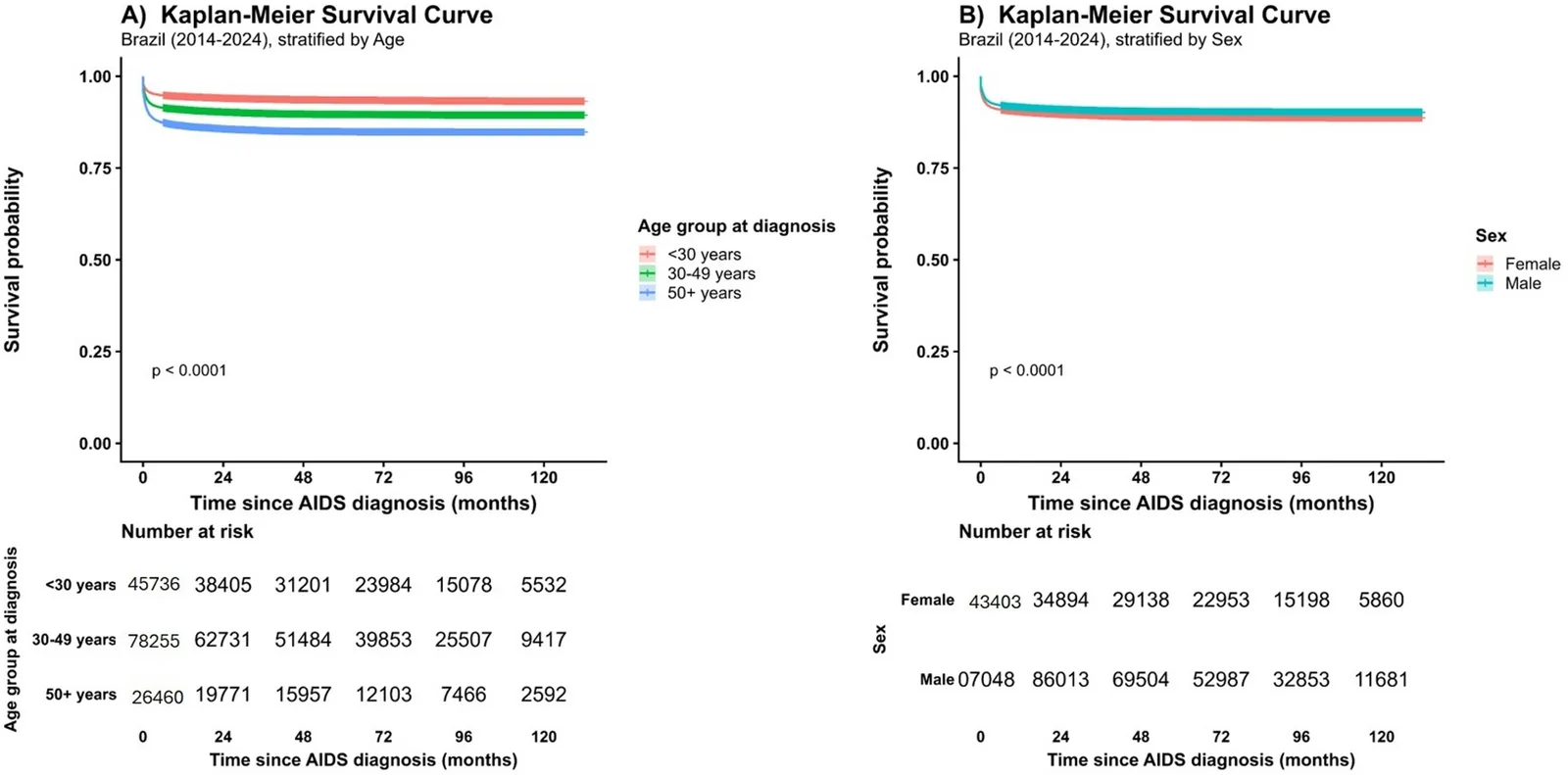
Kaplan-Meier survival curves for patients diagnosed with AIDS in Brazil, 2014–2024. (**A**) Survival curves stratified by age group at diagnosis (< 30 years, 30–49 years, and 50 + years). (**B**) Survival curves stratified by sex. Shaded areas represent 95% confidence intervals. The tables below the plots indicate the number of individuals at risk at different time points in months. P-values were obtained using the log-rank test

Survival analysis was performed among 150,451 individuals with AIDS case notifications and complete information for all covariates included in the model. During follow-up, 14,996 AIDS-related deaths were recorded. Overall survival after AIDS diagnosis remained high throughout the study period. Kaplan-Meier curves showed significant differences in survival probabilities according to both age group at diagnosis and sex (log-rank *p* < 0.0001 for both comparisons; Fig. 6).

When stratified by age group, survival was progressively lower among individuals diagnosed at older ages. At 60 months after AIDS diagnosis, estimated survival was highest among individuals younger than 30 years and progressively lower among those aged 30–49 years and 50 years or older, indicating a clear age-related gradient in AIDS-related mortality (Fig. 6A). When stratified by sex, males showed slightly higher survival than females over follow-up, although the visual separation between curves was modest (Fig. 6B).

### Cox Regression Analysis

In the multivariable Cox proportional hazards model (Table 3), male sex was associated with a lower risk of AIDS-related mortality compared with female sex (AHR = 0.84; 95% CI: 0.80–0.88; *p* < 0.001). Age at diagnosis remained strongly associated with mortality. Compared with individuals younger than 30 years, those aged 30–49 years had a higher risk of AIDS-related death (AHR = 1.42; 95% CI: 1.36–1.49; *p* < 0.001), and this risk was even higher among individuals aged 50 years or older (AHR = 1.95; 95% CI: 1.86–2.05; *p* < 0.001).

**Table 3:** Prognostic factors associated with AIDS-related mortality among AIDS case notifications in Brazil, 2014–2024 (*N* = 150,451). AHR, adjusted hazard ratio; CI, confidence interval; MSM, men who have sex with men. The model included 150,451 individuals with AIDS case notifications and complete information for all covariates, with 14,996 AIDS-related deaths during follow-up

Variable/category	AHR (95% CI)	*p*-value
Sex		
Female	Reference	
Male	0.84 (0.80–0.88)	< 0.001
Age group at diagnosis		
< 30 years	Reference	
30–49 years	1.42 (1.36–1.49)	< 0.001
50 + years	1.95 (1.86–2.05)	< 0.001
Region		
Center-West	Reference	
North	0.87 (0.81–0.93)	< 0.001
Northeast	0.88 (0.83–0.94)	< 0.001
South	0.67 (0.63–0.71)	< 0.001
Southeast	0.68 (0.64–0.72)	< 0.001
Race/skin color		
White	Reference	
Black	0.99 (0.94–1.05)	0.847
Parda	1.05 (1.01–1.10)	0.012
Other/ignored	0.85 (0.75–0.95)	0.005
Education		
Higher education	Reference	
Fundamental or less	2.09 (1.97–2.22)	< 0.001
High school	1.51 (1.42–1.60)	< 0.001
Exposure category		
Heterosexual	Reference	
Injecting drug user	2.83 (2.62–3.06)	< 0.001
Men who have sex with men (MSM)	1.32 (1.27–1.38)	< 0.001

Regional differences were also observed. Compared with the Center-West region, individuals from the North (AHR = 0.87; 95% CI: 0.81–0.93; *p* < 0.001), Northeast (AHR = 0.88; 95% CI: 0.83–0.94; *p* < 0.001), South (AHR = 0.67; 95% CI: 0.63–0.71; *p* < 0.001), and Southeast (AHR = 0.68; 95% CI: 0.64–0.72; *p* < 0.001) had lower risk of AIDS-related mortality. Regarding race/skin color, parda individuals had a slightly higher risk of mortality compared with white individuals (AHR = 1.05; 95% CI: 1.01–1.10; *p* = 0.012), whereas black individuals did not differ significantly from white individuals (AHR = 0.99; 95% CI: 0.94–1.05; *p* = 0.847).

Educational level showed one of the strongest associations with AIDS-related mortality. Individuals with fundamental education or less had more than twice the risk of death compared with those with higher education (AHR = 2.09; 95% CI: 1.97–2.22; *p* < 0.001), while those with high school education also had increased risk (AHR = 1.51; 95% CI: 1.42–1.60; *p* < 0.001). Finally, by exposure category, injecting drug users had the highest risk of AIDS-related mortality (AHR = 2.83; 95% CI: 2.62–3.06; *p* < 0.001), followed by men who have sex with men (AHR = 1.32; 95% CI: 1.27–1.38; *p* < 0.001).

## Discussion

This study analyzed the dynamics of HIV infection and AIDS case notifications in Brazil from 2014 to 2024, showing that the national HIV/AIDS epidemic remains complex, heterogeneous, and strongly shaped by regional and sociodemographic inequalities. Although Brazil is frequently cited as an international reference because of its pioneering policies, including universal and free access to combined antiretroviral therapy (c-ART) since 1996, our findings indicate that these advances have not been equally distributed across all populations and territories [8].

A recent national analysis by Lopes-Araujo et al. (2026) provides an important framework for interpreting our results. The authors showed that, between 2015 and 2024, HIV incidence increased in Brazil, whereas AIDS incidence and AIDS-related mortality declined. This apparent paradox was attributed to improvements in the HIV care cascade, particularly earlier ART initiation and sustained viral suppression. In that study, the proportion of people living with HIV who initiated ART within 30 days after diagnosis increased from 28% to 63%, while ART initiation after more than 6 months decreased from 35% to 10%. Early ART initiation was strongly and negatively correlated with AIDS incidence and AIDS-related mortality [9]. Our study complements this macroepidemiological interpretation by identifying who is being notified as HIV infection or AIDS, where these notifications are concentrated, and which characteristics are associated with AIDS-stage notification and AIDS-related death.

The temporal analysis further illustrates the changing dynamics of HIV/AIDS surveillance in Brazil. HIV infection notifications increased markedly between 2014 and 2017, followed by relative stabilization between 2018 and 2019. This initial increase should not be interpreted exclusively as a rise in HIV transmission, because it may also reflect the progressive consolidation of HIV infection reporting after its incorporation into the national compulsory notification system, as well as the expansion of testing and surveillance capacity. In contrast, AIDS case notifications showed a more consistent downward pattern over most of the study period, suggesting a gradual reduction in progression to AIDS-stage notification in parallel with improvements in diagnosis, treatment access, and continuity of care. This interpretation is consistent with national evidence showing that AIDS incidence and AIDS-related mortality declined in Brazil during a period in which HIV incidence remained stable or increased, a pattern mainly attributed to earlier ART initiation and sustained viral suppression [9].

A clear disruption was observed in 2020, when both HIV infection and AIDS case notifications decreased substantially. This decline should be interpreted with caution, because it likely reflects the indirect effects of the COVID-19 pandemic on access to HIV testing, outpatient care, surveillance routines, and linkage to health services rather than a true abrupt reduction in HIV transmission or AIDS occurrence. In Brazil, Andrade et al. (2023) reported reductions in HIV/AIDS diagnoses during the pandemic period and suggested that delayed diagnosis may have contributed to worse clinical outcomes. Similar disruptions were also described in broader analyses of HIV testing and service delivery during COVID-19, which documented reductions in testing volume, interruptions in routine care, and adaptations in HIV services across different settings [10]. Therefore, the 2020 decline observed in our study is best interpreted as a marker of health system disruption and possible underdiagnosis or undernotification.

After 2020, notifications increased again, particularly for HIV infection, reaching high levels in 2022 and 2023. This rebound suggests a partial recovery of testing, diagnostic access, and notification capacity after the acute phase of the pandemic. Brazilian studies have shown that COVID-19 affected HIV care indicators, including service attendance, laboratory monitoring, and treatment continuity, with impacts varying according to age, gender, and population group [11, 12]. In this context, the recovery observed after 2020 may reflect the gradual resumption of HIV-related services and surveillance activities. However, the sharp decline observed in 2024 should also be interpreted cautiously. Although 2024 data were included in the analysis, recent notification years may be more susceptible to delayed data entry, late consolidation, and administrative updating in national surveillance systems. Thus, the 2024 decrease may represent either a real short-term decline in notifications or residual underreporting related to data consolidation.

Taken together, these temporal findings indicate that the decline in AIDS case notifications over time is compatible with improvements in the HIV care cascade and reduced progression to AIDS-stage notification. Conversely, fluctuations in HIV infection notifications appear to be strongly influenced by testing availability, surveillance practices, and health system disruptions. This distinction is important because notification trends do not necessarily reflect only changes in transmission dynamics. They also reflect the capacity of the health system to diagnose, report, and retain individuals in care. Therefore, maintaining HIV testing, prevention, linkage to care, and c-ART continuity during public health emergencies is essential to avoid delayed diagnosis and prevent progression to AIDS-stage notification.

The descriptive analyses showed that the largest absolute numbers of notifications remained concentrated in the most populous states, particularly São Paulo, Rio de Janeiro, Rio Grande do Sul, and Minas Gerais. This pattern is consistent with the historical concentration of the epidemic in major urban and demographic centers in the South and Southeast regions [5]. However, when population-adjusted rates were considered, important regional pockets of higher relative intensity emerged, especially in Rio Grande do Sul and in parts of the North region, such as Roraima and Amapá. Therefore, absolute notification volume and population-adjusted rates captured complementary aspects of the epidemic.

In this sense our spatial analyses reinforced this interpretation. Global Moran’s I showed significant positive spatial autocorrelation, and the LISA analysis identified significant High-High clusters, mainly in the South and Southeast, with additional hotspots in selected municipalities from the North, Northeast, and Center-West regions. This pattern is consistent with previous spatial analyses showing that the epidemic has historically followed urban corridors and transportation routes, while also expanding into less central areas over time [13]. The absence of significant Low-Low clusters suggests that HIV/AIDS notifications remain broadly distributed across Brazil, although with variable local intensity. This finding supports the need for geographically tailored strategies, especially in municipalities identified as hotspots, where testing, linkage to care, ART retention, and prevention actions should be strengthened [5].

These spatial findings also fit within the broader process of regional redistribution of the epidemic. Previous time-series studies have documented declines in AIDS mortality in the South and Southeast, alongside increasing or persistently high rates in the North and Northeast [5, 6, 14]. Thus, the present findings suggest a transitional pattern: the largest absolute number of notifications remains concentrated in major demographic centers, while the relative intensity of the epidemic and vulnerability to worse outcomes increasingly affect areas with more limited access to specialized services. This reinforces the argument that the Brazilian response cannot rely only on national-level indicators, because local inequalities may persist even when aggregate outcomes improve.

Regarding the sociodemographic profile of notifications our results showed clear differences between HIV infection and AIDS case records. HIV infection notifications were concentrated among younger adults, especially men aged 20 to 29 years, whereas AIDS case notifications were more frequent among individuals aged 30 to 49 years and older [15, 16]. This difference may reflect both earlier detection among younger populations and the longer natural history of untreated or insufficiently treated HIV infection among individuals notified at the AIDS stage. In the logistic regression model, older age was strongly associated with AIDS-stage notification, with progressively higher odds across age groups. This finding is consistent with evidence that older adults may have lower perceived risk, lower testing frequency, and delayed clinical suspicion, which can contribute to more advanced disease at notification [17, 18].

This age-related pattern also appeared in the survival analysis. Older age at AIDS diagnosis was one of the strongest predictors of AIDS-related mortality. Individuals aged 50 years or older had almost twice the risk of death compared with those younger than 30 years. Similar age gradients have been reported in Brazilian studies describing increasing AIDS mortality among older adults and higher mortality rates in age groups above 50 years [5, 6, 14]. These findings may be explained by delayed diagnosis, comorbidities, immunosenescence, lower CD4 recovery after ART initiation, and reduced inclusion of older adults in routine HIV testing strategies.

Accordingly, the study by Ke et al. (2023), conducted in Liuzhou, China, strengthens this interpretation from a survival perspective. In that retrospective cohort, survival improved across successive treatment guideline periods as ART initiation became earlier and less dependent on low CD4 thresholds. The authors also found that older age at diagnosis, lower educational level, lack of ART, and lower baseline CD4 counts were associated with mortality [19]. Although the epidemiological context differs from Brazil, this study supports a mechanism that is highly relevant to our findings: survival improves when HIV is detected and treated earlier, whereas older age and social vulnerability continue to increase the risk of death. Because SINAN does not include CD4 count, viral load, ART initiation date, or adherence information, our study could not directly test this pathway. Nevertheless, the association between older age, AIDS-stage notification, lower education, and mortality is consistent with this biological and health service mechanism.

Educational level was one of the strongest sociodemographic determinants of AIDS-related mortality. Individuals with fundamental education or less had more than twice the risk of death compared with those with higher education, and those with high school education also had increased risk. This finding agrees with previous studies identifying low education as a marker of delayed diagnosis, reduced health literacy, lower access to prevention, and poorer continuity of care [18, 20, 21]. Education should therefore be interpreted as a structural marker, reflecting unequal access to information, timely testing, specialized services, and sustained treatment.

Race/skin color also contributed to differences in mortality. Parda individuals showed a slightly higher adjusted risk of AIDS-related death compared with white individuals, while black individuals did not differ significantly after adjustment. This result should be interpreted cautiously because race/skin color in Brazilian surveillance systems is affected by self-classification, missingness, and regional demographic composition. Even so, the finding is consistent with recent national analyses showing a growing proportional burden of AIDS-related deaths among parda individuals, especially in the North and Northeast regions [5, 6]. In this context, race/skin color likely reflects social vulnerability, territorial inequality, and unequal access to timely and continuous HIV care rather than a biological determinant.

Our findings also showed that exposure category identified populations that remain vulnerable. Injecting drug users had higher odds of AIDS-stage notification and the highest risk of AIDS-related mortality in the Cox model. MSM also showed increased odds of AIDS-stage notification and higher AIDS-related mortality compared with heterosexual exposure. These findings are consistent with evidence that key populations may face overlapping barriers, including stigma, discrimination, reduced access to health services, delayed testing, and interruptions in care [22]. They also reinforce the conclusion of Lopes-Araujo et al. (2026) that persistent HIV incidence in Brazil requires intensified prevention campaigns, stigma reduction, and targeted interventions in high-risk populations [9].

Regarding the association between male sex and lower AIDS-related mortality, this finding should be interpreted with caution. Men accounted for most notifications, indicating greater notification burden. However, in the adjusted Cox model, male sex was associated with slightly lower mortality compared with female sex. This does not mean that men are less vulnerable to HIV infection. Rather, it suggests that mortality after AIDS notification is influenced by a combination of age, education, exposure category, race/skin color, region, and access pathways [23, 24]. The finding may also reflect the effect of targeted testing and treatment strategies that have reached some male populations more effectively in recent years. This interpretation is compatible with broader analyses showing declines in AIDS mortality for both men and women in Brazil, while also highlighting that sex-specific patterns may change depending on the period and covariates considered [5].

Taken together, our findings show that the current HIV/AIDS scenario in Brazil is not defined only by the overall volume of notifications, but by the unequal distribution of AIDS-stage notification and AIDS-related mortality across populations and territories. By combining descriptive, spatial, logistic regression, and survival analyses, this study highlights that older adults, people with lower education, injecting drug users, MSM, parda individuals, and municipalities located in significant spatial clusters remain central to the persistence of severe HIV/AIDS outcomes. These results indicate that the next step in the Brazilian HIV response should focus on reducing the gap between national-level progress and local-level vulnerability, ensuring that early testing, linkage to care, ART initiation, and long-term follow-up effectively reach the groups and areas where the burden remains concentrated.

## Limitations

This study has limitations inherent to the use of secondary surveillance data. SINAN records may contain missing, incomplete, or inconsistent information, especially for education, race/skin color, and exposure category. The complete-case approach used in the multivariable models may have introduced selection bias, since individuals with complete records may differ systematically from those with incomplete data. Therefore, the regression results should be interpreted in light of this limitation.

In addition, the analyses were based on notifications rather than direct measures of HIV incidence or prevalence. Although SINAN is the main national surveillance system for HIV/AIDS in Brazil, underreporting, duplicate records, and differences in reporting quality across regions may have affected the observed patterns. The operational definition of AIDS-stage notification was based on the structure of the notification databases and should not be interpreted as a clinical definition based on CD4 count, viral load, or time since infection.

Another important limitation is the absence of clinical variables such as CD4 count, viral load, ART initiation date, adherence, comorbidities, and retention in care.

This prevented direct evaluation of mechanisms related to early ART initiation and viral suppression, which have been shown to explain reductions in AIDS incidence and mortality in Brazil [9]. Similarly, although international evidence indicates that baseline CD4 count and ART initiation are key determinants of survival [19], these variables were not available in the SINAN database used in the present study. Finally, because the proportional hazards assumption showed evidence of violation, adjusted hazard ratios from the Cox model should be interpreted as average associations over follow-up rather than constant effects over time.

## Conclusion

The HIV/AIDS epidemic in Brazil remains characterized by both progress and persistent inequality. Between 2014 and 2024, HIV infection notifications predominated over AIDS case notifications, while AIDS-stage notification and AIDS-related mortality were unevenly distributed across sociodemographic groups and geographic areas. These findings support recent national evidence showing that persistent or increasing HIV detection can coexist with declining AIDS incidence and mortality, likely because of expanded testing, earlier ART initiation, and sustained viral suppression.

However, the benefits of these advances are not uniformly distributed. Older adults, people with lower education, injecting drug users, MSM, parda individuals, and municipalities located in significant spatial clusters remain priority groups for public health action. Strengthening the HIV care cascade in Brazil requires targeted and locally adapted strategies focused on timely testing, early linkage to care, rapid ART initiation, retention in treatment, and stigma reduction. To further reduce AIDS-stage notification and AIDS-related mortality, national progress must be translated into effective interventions for the populations and territories where vulnerability persists.

## Data Availability

The raw SINAN .dbc files are publicly available from DATASUS. The scripts used for data loading, processing, analysis, and visualization are available at: https://github.com/Bruno-4lmeida/SINAN_HIV.
